# Automated Electronic Health Record to Electronic Data Capture Transfer in Clinical Studies in the German Health Care System: Feasibility Study and Gap Analysis

**DOI:** 10.2196/47958

**Published:** 2023-08-04

**Authors:** Christian Mueller, Patrick Herrmann, Stephan Cichos, Bernhard Remes, Erwin Junker, Tobias Hastenteufel, Markus Mundhenke

**Affiliations:** 1 Bayer Vital GmbH Leverkusen Germany; 2 Bayer AG Berlin Germany; 3 Bayer AG Wuppertal Germany; 4 Alcedis GmbH Gießen Germany; 5 Qurasoft GmbH Koblenz Germany

**Keywords:** digital transformation, automated data transfer, electronic medical record, electronic data capture, EDC, data transfer, electronic health record, EHR, digital transfer, barrier, clinical practice, EHR2EDC, health care system

## Abstract

**Background:**

Data transfer between electronic health records (EHRs) at the point of care and electronic data capture (EDC) systems for clinical research is still mainly carried out manually, which is error-prone as well as cost- and time-intensive. Automated digital transfer from EHRs to EDC systems (EHR2EDC) would enable more accurate and efficient data capture but has so far encountered technological barriers primarily related to data format and the technological environment: in Germany, health care data are collected at the point of care in a variety of often individualized practice management systems (PMSs), most of them not interoperable. Data quality for research purposes within EDC systems must meet the requirements of regulatory authorities for standardized submission of clinical trial data and safety reports.

**Objective:**

We aimed to develop a model for automated data transfer as part of an observational study that allows data of sufficient quality to be captured at the point of care, extracted from various PMSs, and automatically transferred to electronic case report forms in EDC systems. This required addressing aspects of data security, as well as the lack of compatibility between EHR health care data and the data quality required in EDC systems for clinical research.

**Methods:**

The SaniQ software platform (Qurasoft GmbH) is already used to extract and harmonize predefined variables from electronic medical records of different Compu Group Medical–hosted PMSs. From there, data are automatically transferred to the validated AlcedisTRIAL EDC system (Alcedis GmbH) for data collection and management. EHR2EDC synchronization occurs automatically overnight, and real-time updates can be initiated manually following each data entry in the EHR. The electronic case report form (eCRF) contains 13 forms with 274 variables. Of these, 5 forms with 185 variables contain 67 automatically transferable variables (67/274, 24% of all variables and 67/185, 36% of eligible variables).

**Results:**

This model for automated data transfer bridges the current gap between clinical practice data capture at the point of care and the data sets required by regulatory agencies; it also enables automated EHR2EDC data transfer in compliance with the General Data Protection Regulation (GDPR). It addresses feasibility, connectivity, and system compatibility of currently used PMSs in health care and clinical research and is therefore directly applicable.

**Conclusions:**

This use case demonstrates that secure, consistent, and automated end-to-end data transmission from the treating physician to the regulatory authority is feasible. Automated data transmission can be expected to reduce effort and save resources and costs while ensuring high data quality. This may facilitate the conduct of studies for both study sites and sponsors, thereby accelerating the development of new drugs. Nevertheless, the industry-wide implementation of EHR2EDC requires policy decisions that set the framework for the use of research data based on routine PMS data.

## Introduction

The digital transformation of the German health care system currently taking place opens up completely new opportunities but also creates challenges for the future. Data quality for different applications needs to be considered and managed accordingly. The global pathway to market authorization is highly regulated in drug development and follows the highest standards for curated data quality. Utilization of routine health care data for clinical research in Europe is increasingly coming into focus and offers considerable potential to accelerate development of innovative therapies and facilitate monitoring of their implementation in everyday clinical practice; this can contribute considerably to improving the quality of health care. Today, the standard for an investigator participating in primary data collection is double documentation in practice management systems (PMSs) or hospital information systems (HISs), as well as in electronic data capture (EDC) systems, to comply with the needs of the pharmaceutical industry and the requirements of regulatory bodies. In parallel, the European Health Data Space (EHDS) legislation addresses the requirements for the use of primary data (the MyHealth@EU program) to improve health care in routine clinical practice and strengthen interoperability between the different European health care systems; it also addresses the use of secondary data for research purposes (the HealthData@EU program) [1-3]. This provides access to health data for research on new prevention strategies as well as for disease diagnosis and treatment. Both approaches have to ensure patient control over their personal data [4]. However, little thought has yet been given as to how routine clinical data can be utilized in pseudonymized form—as is required in clinical research as part of safety and efficacy studies for the development and approval of new drugs.

Apart from many other challenges to the implementation and active use of the EHDS, such as interoperability and data protection, the main issues remain access to, usability of, and quality of routine clinical data for the research purposes of sponsors such as the pharmaceutical industry. In particular, issues of data security, the lack of compatibility between health data captured in electronic health records (EHRs) in clinical practice and data required in EDC systems for clinical research, and the efficient transfer from EHRs to EDC (EHR2EDC) need to be resolved. In this context, the pharmaceutical industry can make important contributions due to its current professional management of data and information resources for pseudonymized real-world data collection based on routine documentation in HISs or PMSs, as well as anonymized secondary data analysis. Both have been standard practice in the pharmaceutical industry for decades.

The next step is automation of the data transfer process with secondary data use in the EHDS. The aim is to capture data with sufficient quality at the point of care and automatically transfer these data to electronic case report forms (eCRFs) in EDC systems. The overarching benefits for investigators are reduced effort and saved resources and costs with the accuracy of data maintained. In order to realize this goal in the near future, we set up a feasibility study for an automated data transfer process within the prospective, noninterventional FINE-REAL study, a study providing insights into the use of the mineral corticoid receptor antagonist finerenone in a routine clinical setting in patients with diabetes and chronic kidney disease [5].

The aim of this paper is to describe the architecture of this automated process and the issues and challenges that had to be considered during its design. In addition, future opportunities and challenges related to automated health data transfer will be discussed, associated regulatory implications will be outlined, and adaptations for data documentation in routine clinical practice and clinical study design will be proposed to facilitate effective use of health care data for clinical research in the near future.

## Methods

### Ethical Considerations

Ethical approval for this study was granted by the ethics committee of the Friedrich-Alexander-University Erlangen on August 16, 2022 (22-260_1-NIS).

### Objectives

In this collaboration, Bayer, the sponsor of the FINE-REAL study, contracted study sites and provided protocol and informed consent forms to these study sites. Alcedis GmbH is the provider of the eCRFs as part of the AlcedisTRIAL EDC system, and Qurasoft GmbH provides middleware (SaniQ) that connects Compu Group Medical (CGM)–hosted PMSs with the EDC system. The aim was to demonstrate the feasibility of automated data transfer from EHRs in different CGM-hosted PMSs at the participating study sites into the already existing eCRF system of the FINE-REAL study. Evaluation of automated data transfer efficiency and head-to-head comparison of documentation efforts between conventional (manual) and automated data transfer will be based on predefined key performance indicators (KPIs), summarized in Textbox 1.

**Key performance indicators.**

**Key performance indicators**

- Time spent for documentation (per patient, per data item, and per form)
- Satisfaction of documentalists (questionnaire)
- Percentage of variables that are transferable automatically
- Percentage of automatically transferable variables actually available in the electronic data capture system
- Number of queries

### Status Quo and Obstacles

Although many physicians’ offices and hospitals already use EHRs within their PMSs, most of these are not interoperable with EDC systems. A lack of interfaces and accepted standards on both sides degrades health care professionals to the level of medieval copyists (monks who copied the Bible from previous copies) when they manually transfer data already entered into an EHR into the EDC system. This is not only time-consuming and resource-intensive, but also carries the risk of transmission errors. However, automated electronic data transfer still faces technological obstacles, mainly related to data format and data quality.

### Variability of Systems and Lack of Web Connectivity

The challenge starts with the very diverse landscape of commonly used PMSs and subsystems in Germany, all of which must be approved by the Kassenärztliche Bundesvereinigung (the National Association of Statutory Health Insurance Physicians) [6]. Although certain systems are often used preferentially within a medical discipline, there is no standard, predominantly used provider across all medical specialties. PMSs are designed to operationally support health care professionals in managing patient data, scheduling appointments, prescribing medications, and billing statutory health insurance for health care services, but they are not designed to support clinical studies or perform research with real-life data. The bottleneck for automated data transfer for clinical studies involving multiple centers from potentially various disciplines is therefore accessing and extracting data from these different systems. To further complicate the situation, some of the PMSs and HISs currently in widespread use date back to the 1980s and are not at all interoperable with HL7 (Health Level Seven International), the widely accepted standard for data exchange between health care software systems, or its most recent enhancement FHIR (Fast Healthcare Interoperability Resources) [7,8]. FHIR has emerged as an effective standard for health care data interoperability that enables seamless exchange of EHRs and facilitates the integration of various health care systems; however, it is not yet widely implemented in the ambulatory sector.

### Data Quality and Data Standards

On the other end of the data chain, clinical studies and noninterventional studies of the pharmaceutical industry—and, accordingly, the corresponding eCRFs of EDC systems—have to comply with the requirements for submission of clinical study data and safety reporting of the regulatory authorities. In addition to those standards, the EDC system needs to be compliant to the US Food and Drug Administration (FDA) Code of Federal Regulations (CFR), Title 21, Part 11, and the European Union EudraLex V4, Annex 11, in order to make the electronic records be equivalent to the traditional paper records [9-12]. This entails a full validation of the system, a backup and restore process, change and configuration management, access control, data printout functionality, the maintenance of an audit trail, built-in data checks, and the presence of an electronic signature for the investigator to confirm correctness and completeness of the study data.

In contrast, PMSs can be individually customized to the specific demands of a practice, often making an automated transfer of these data impossible. In addition, subsystems such as laboratory information systems and medication systems must also be taken into account for automated data transfer, as these contain laboratory values and medication information that are often particularly important for clinical studies. Standardization of terminology and lab values within these systems is missing for the time being. Furthermore, even if data are regularly updated in the PMS, only the current status of a variable can be extracted for study documentation, not its development over time, as required for many studies. For example, in the context of a diabetes study, only the current weight can be captured from an EHR in the PMS, not its longitudinal development over the course of the study. Finally, there is huge discrepancy between data documentation in EHRs and the high level of granularity required in eCRFs. For example, comorbidities are usually documented via *International Statistical Classification of Diseases and Related Health Problems, Tenth Revision (ICD-10)* codes; however, more detailed information regarding potential subtypes might be required for the EDC and subsequent processes, such as medical coding.

### Digital Architecture of Automated Data Transfer

**Figure 1:**
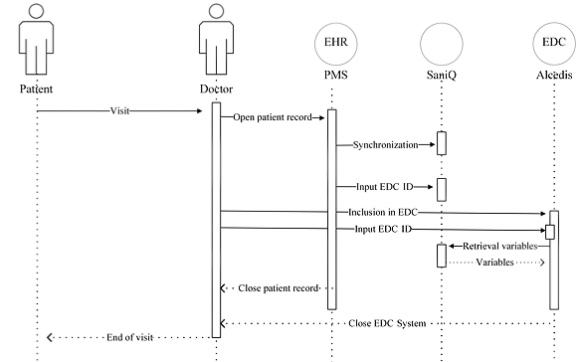
Digital architecture for automated data transfer as one option of the FINE-REAL study. Data are extracted and harmonized from the EHRs of various PMSs at the study centers using SaniQ software and transferred to the AlcedisTRIAL EDC system. Solid arrows indicate a synchronous request (arrow pointing to the right) or query (arrow pointing to the left); the dashed arrows indicate an asynchronous request or query. EDC: electronic data capture; EHR: electronic health record; PMS: practice management system.

We tackled these issues by extracting and harmonizing predefined variables from EHRs in various PMSs as well as was possible at the included study centers, using the SaniQ software platform (Qurasoft GmbH). From there, data were automatically transferred to the validated AlcedisTRIAL EDC system (Alcedis GmbH) for data capture and management. This process is fully server-based, that is, all program processes are executed centrally on a web or database server (Figure 1).

The SaniQ software enables the extraction, as well as the harmonization, of data from different CGM-hosted PMSs via an interface. By connecting with the PMS, the investigator is able to select single patient records to be transferred to SaniQ, a cloud-based relational database. The a priori defined study variables that are eligible to be transferred to the EDC allow partial documentation of vital parameters such as age, weight, or comorbidity values. The automatic EHR2EDC update occurs overnight; however, a real-time update of the EDC system can be requested manually following each data entry in the EHR.

The AlcedisTRIAL software is developed and set up using GAMP (good automated manufacturing practice) standards and complies with FDA Title 21 CFR Part 11 and European Union EudraLex V4 Annex 11 regulations [13]. Processed data are stored in a relational and access-restricted database with specification and implementation of the database structure based on the requirements of the particular study. Study-specific eCRFs are used to document patient data, including live validation of eCRFs during data capture. Both access rights and dedicated rights of the corresponding CRUD (create, read, update, and delete) functions are managed via a role-based access system. Once data is transferred from SaniQ to AlcedisTRIAL, the last step of mapping occurs—the manual documentation of further variables into the EDC.

### Automatically Transferable Variables

Variables that are automatically transferable from SaniQ to the Alcedis EDC system were determined jointly by Bayer, Alcedis, and Qurasoft based on the existing eCRFs of the FINE-REAL study. The eCRFs contain 13 forms with 274 variables. Of these, 5 forms with 185 variables contain 67 automatically transferable variables (67/274, 24% of all variables and 67/185, 36% of eligible variables). All automatically transferable variables are exclusively editable in the source data system, that is, the EHR of the respective PMS, while all variables that are not automatically transferable are editable in the EDC system. Laboratory values provided by laboratory information systems (LISs) to investigators can be transferred automatically as well, if the LIS is connected directly with SaniQ. Table 1 provides an overview of the automatically transferable variables within the FINE-REAL study. Detailed information on the automatically transferable data on comorbidities per *ICD-10* code is available in Multimedia Appendix 1. The end-to-end data transfer from the PMS via SaniQ to EDC is organized on a daily basis with regular data transfers if new predefined variables are available within the PMS, but also via push transfers to create visits in the EDC system.

**Table 1:** Overview of automatically transferable variables within the FINE-REAL-study.

Category	Variables
General data	Year of birth Age Gender Weight (kg) Height (cm)
Laboratory values	Query whether laboratory software is used and accordingly connected via laboratory data transfer
Comorbidities	Comorbities? (yes/no) Comorbitity according to *International Statistical Classification of Diseases and Related Health Problems, Tenth Revision* codes^a^
Medication	Any finerenone prescribed? (yes/no) Pharmaceutical central number (*Pharmazentralnummer*) of other prescribed medications Start date of prescribed medications with frequency and planned dose

^a^For detailed information, see Multimedia Appendix 1.

### Administrative and Scheduling Workflows

After signing the contract with the sponsor for participation in the FINE-REAL study, investigators have to choose between manual (conventional) data documentation and automated data transfer to allow comparison of the 2 approaches. In case of automated data transfer, a corresponding contract adjustment will be made between the sponsor and the investigator. Data privacy–compliant use of patients’ personal data in accordance with the General Data Protection Regulation (GDPR) is ensured by data processing agreements and obtaining consent of participating patients to the transfer of data to a third-party system [14]. Moreover, data workflow and cloud storage are explained in the consent form. All investigators receive access and training in the EDC system of the study. After successfully passing a knowledge test, data documentation in the EDC system can start (the investigator is ready to enroll).

For this purpose, the investigator manually adds patients to the EDC system, which automatically generates a unique, pseudonymous number for each patient that is exclusively used for the FINE-REAL study EDC system. Both at the SaniQ level and in the EDC system, reidentification of a patient is only possible for authorized site personnel via the patient identification list, which is kept strictly confidential at the study site.

In parallel, Qurasoft is creating an individual SaniQ practice domain with the investigator’s key data so that eligible study sites (centers with a CGM-hosted PMS) can gain access to the SaniQ system. After the main user generates an individual password for the system following an automatic invitation email, the SaniQ practice system is accessible with all its functions. Subsequently, a personal identifier (called the doc ID) can be generated within the account menu, which is required to establish the connection with the PMS for data transfer. After entering the doc ID in the PMS, the investigator receives a confirmation email that a connection between their SaniQ practice domain and the PMS has been established, which can be disconnected at any time by the SaniQ practice domain. The last step to set up automatic transfer is the entry of the patient ID generated within AlcedisTRIAL into the SaniQ system to ensure the matching of each patient. Synchronization between the SaniQ practice domain and the PMS occurs automatically overnight and can be initiated manually each time a patient’s EHR is opened and processed (Figure 1).

As soon as the investigator documents data during the baseline visit in the PMS, transfer of automatically transferable variables occurs overnight and the corresponding visit is created in the EDC system. The investigator then completes the forms in the EDC system by including manually editable data and finalizes the baseline visit. During the course of the study, patients are routinely treated and the treatment, including laboratory values, is documented in the EHR of the PMS as usual. Each follow-up visit is created in the eCRF either by the physician or automatically by the presence of data automatically transferred from the PMS. Adverse events, queries, and signature requests have to be documented manually by the investigator in the EDC system without electronic reconciliation in the PMS.

## Results

This model for automated data transfer has the potential to bridge the current gap between clinical practice data capture at the point of care and the data sets required by regulatory agencies, enabling GDPR-compliant automated EHR2EDC data transfer. It addresses feasibility, connectivity, and system compatibility of currently used CGM-hosted systems in health care and clinical research and is therefore directly applicable. In December 2022, the first patient was enrolled in the observational FINE-Real study in a center with conventional data transfer. Automated data transfer will be activated at 5 study sites beginning in May 2023 and will last until the end of the FINE-REAL study. The added value of automated data transfer will be measured according to the predefined KPIs in this feasibility study.

## Discussion

### Principal Findings

The use case described here demonstrates the feasibility of automated EHR2EDC data transfer for clinical research to address the needs of the pharmaceutical industry. We hope our work will contribute to collaborative solutions as the German health care system enters a digital transition phase with regard to efficient and GDPR-compliant use of health data for clinical research and accelerate a secure digital transformation.

This model for automated data transfer bridges the currently existing gap between data collection in clinical practice at the point of care and the data sets required by regulatory authorities through automated data transfer, enabling efficient use of clinical data for research purposes.

A key benefit of the automated data transfer model presented here is the fact that it addresses the feasibility, connectivity, and compatibility of the systems still predominantly used in German health care and clinical research and is therefore directly applicable: the model is connectable to various CGM-hosted PMSs currently used in health care and to clinical research activities demanded by the authorities that require pseudonymized primary data collection (eg, postauthorization safety studies). To ensure that the data are accepted by the authorities, transparent and accurate presentation of the data and data transfer are required, as is a high level of control by the authorities, all of which is met in our use case.

Another key advantage is the ability to collect data prospectively over time. In contrast to systems that offer retrospective or cross-sectional insights into the medical data of specific PMSs at specific time points in the past (eg, studies that use post hoc secondary analyses of anonymized data) the digital architecture of the data transfer described here also enables prospective automated data extraction of pseudonymized data from routine health care over a time course into the future. This is a crucial aspect for conducting clinical trials, for gaining relevant insights, especially into chronic diseases, and for assessing the efficiency of treatment management in routine clinical practice.

In addition, data utilization always occurs with patient consent and in compliance with the GDPR. In the future, the data transfer model presented here could be imagined to include the *elektronische Patientenakte* (ePA; the German term for EHRs) as a source system for pseudonymized routine data utilization; the system could thus make a significant contribution to the increased use of health data envisioned within the EHDS. Section 363 (8) of the German Social Security Act V (Sozialgesetzbuch V) on the processing of data from EHRs for research purposes already addresses the possibility of each patient using an ePA to transmit health data to researchers—not just to the research data center (*Forschungsdatenzentrum*)—on the basis of an individual declaration of consent. This is a legally, but currently operationally incompatible way to provide data for research studies sponsored by the pharmaceutical industry. If this were feasible from an interoperability aspect, it would allow patients to make free and informed decisions if and to what extent they want to support research projects with their ePA data.

Overall, the automated data transfer model presented here enables an efficient and secure flow of data from the treating physician in routine clinical practice via clinical research to regulatory authorities. At the same time, the documentation effort is likely to be reduced considerably, as automatically transferable variables are to be documented at only one location (in the PMS). It is anticipated that this reduced documentation burden may promote the willingness of health care professionals to participate in clinical studies. In any case, in a time of shortages of health care professionals and simultaneously increasing numbers of patients—especially in the field of age-related chronic diseases—our system offers the opportunity to focus attention on the meaningful aspects of clinical studies, rather than simple data transcription, as is currently still often the case in clinical studies.

Furthermore, the automated data transfer described here ensures the high quality of the collected data and should reduce queries and monitoring efforts. This may reduce costs and improve study planning, giving the pharmaceutical industry a greater stimulus to develop new drugs more quickly.

The proportion of variables for the clinical study that could be transferred automatically (24% of all variables) was somewhat lower in our model than in other approaches for automated data transfer [15,16]. Due to differing approaches toward automated data transfer, however, solely considering the proportion of automatically transferable variables is inadequate and resembles an apples-to-oranges comparison. Consideration must also be given to the extent to which automated data transfer is connectable to existing systems in clinical practice to enable broad participation of physicians’ offices and hospitals as potential study sites. Moreover, it has to be noted that in this pilot project for automated data transfer, the eCRFs of the clinical study had already been determined. This raises important questions for the requirements for health care data utilization in clinical research in the future—in terms of routine data collection, clinical study design, and the need for study-specific modules for automated data capture in fully interoperable systems in routine practice with standard terminology.

### Future Implications

In the future, eCRFs might be designed to be 100% powered by health care data. This would require a “leaner” clinical study design with a more precise research question aligned to available health care data. It might also be useful to divide clinical studies into manageable “parts” that could be implemented more quickly. Alternatively, a hybrid model for health care data utilization combining automated and manual documentation, as described here, may continue to be used. Such a hybrid approach would leverage the power of automation and the qualifications of pretrained investigators, who know their patients best. Regardless of which path is taken, it seems imperative to improve the quality of data collection at the point of care so that health care data sets can actually be used for clinical research and regulatory purposes in the future.

The Observational Health Data Science and Informatics (OHDSI) community provides an open-source software portfolio and methods for data standardization and analysis that include the Observational Medical Outcomes Partnership (OMOP) common data model. As part of the German Medical Informatics Initiative, a collaboration between HL7 and the OHDSI community was announced in March 2021. The goal is to create a single common data model that integrates HL7 FHIR and OMOP and focuses on open-source software to enable data integration and distributed analyses. Just as our EHR2EDC approach is applicable to the outpatient sector, the OMOP common data model seems to be a starting point for automating the transfer of study data in the hospital setting.

Furthermore, as the HL7 FHIR takes a modular approach and represents granular health care data (eg, heart rate, comedication, intolerances) as independent modular entities, its widespread implementation is of utmost importance for automatic data transfer to ensure correct variables can be transferred with EHR2EDC [17,18].

### Good Documentation Practice in Clinical Trials

Currently, the treatment of patients is documented by the physician either as free text or in prespecified fields in the EHR in the PMS; in the future, this will be based on FHIR standards. In clinical trials and noninterventional studies, however, the pharmaceutical industry serves the data formats required by the regulatory authorities and has aligned the data structure in eCRFs to provide study data tabulation model (SDTM) data sets at the end of the study as required by authorities. To overcome this gap, standardized routine health care documentation is needed to enable collection and automated transfer of as much deeply structured data as possible for its use in clinical research. This would require bringing data capture forward from the time of eCRF documentation to the time of primary documentation in the EHR. Put simply, it would be reasonable for routine documentation at the point of care to meet the requirements of study documentation demanded by regulatory authorities. Furthermore, the implementation of a standardized interface directly between EHRs and the EDC system, without any middleware as in the process described here, would also result in a leaner dataflow process, as already envisioned in other use cases within the collaborative health data space hosted by Bund der Deutschen Industrie (BDI) [19].

To be able to use routine documentation at the point of care for study documentation in the future, good documentation recommendations—similar to guideline treatment recommendations—would be required, that is, precise definitions of indication-specific variable sets would be required that contained all variables necessary for structured documentation in a basic data set of a specific disease; this would enhance already-known deliverables, like disease management program (DMP) documentation indicating special impacts on scale and budget. This approach would also comply with the recommendations of the German Society for Internal Medicine (Deutsche Gesellschaft für Innere Medizin; DGIM) on the contents of the ePA [20], make an important contribution to better patient care, and facilitate the automated transfer of deeply structured data with a high level of granularity from routine clinical practice to clinical research. Beyond technical issues, it will also be necessary to clarify and adapt the way physicians are remunerated for their increased documentation efforts in these kinds of sponsored studies.

As part of its digitization strategy, the German Federal Ministry of Health aims to connect Germany to the EHDS and strengthen the research data landscape in Germany [21]. According to a publication in March 2023, a digital research pseudonym is planned to be introduced by stages in health care registers as well as in routine and study data, thus enabling the use of health care data for research purposes [22]. In addition, in the medium term, it is planned to also link this research pseudonym to ePA data and provide these data to research data centers for secondary analyses. However, it remains an open question as to how existing data collection activities in clinical trials and observational studies can be systematically connected with planned digital research activities. The model for automated data transfer presented here could help enable automated, GDPR-compliant, and secure transfer of data not only with EHR2EDC but also ePA to EDC and thus permit the utilization of clinical routine data for research purposes. In terms of technical development, the first important milestone on the way to automated data transfer will be to achieve core functionality, that is, to make structured data from routine clinical practice usable for clinical research. As a next step, enhanced functionality might be achieved by using natural language processing to make information from the free-text fields accessible for automated data transmission.

### Conclusion

The use case presented here indicates that a secure, privacy-protecting, consistent, and automated data journey from the treating physician to the regulatory authority is feasible. This may contribute to the efficient use of health care data for clinical research, create new capacities, and facilitate the implementation of studies for both study sites and sponsors, such as the pharmaceutical industry. This should increase the willingness to implement clinical studies and accelerate the development of new drugs. Implementation requires policy decisions that set the framework for research data utilization based on routine PMS data.

## Appendix

Multimedia Appendix 1 Comorbidities by *International Statistical Classification of Diseases and Related Health Problems, Tenth Revision (ICD-10)* codes.

## Abbreviations

BDI: Bund der Deutschen Industrie
CFR: Code of Federal Regulations
CGM: Compu Group Medical
CRUD: create, read, update, and delete
DGIM: Deutsche Gesellschaft für Innere Medizin (German Society for Internal Medicine)
DMP: disease management program
eCRF: electronic case report form
EDC: electronic data capture
EHDS: European Health Data Space
EHR: electronic health record
EHR2EDC: electronic health record to electronic data capture
ePA: elektronische Patientenakte (German term for electronic health record)
FDA: US Food and Drug Administration
FHIR: Fast Health care Interoperability Resources
GAMP: good automated manufacturing practice
GDPR: General Data Protection Regulation
HIS: hospital information system
HL7: Health Level Seven International
ICD-10: *International Statistical Classification of Diseases and Related Health Problems, Tenth Revision*
KPI: key performance indicator
LIS: laboratory information system
OHDSI: Observational Health Data Science and Informatics
OMOP: Observational Medical Outcomes Partnership
PMS: practice management system
SDTM: study data tabulation model
