# Supplementary material for: Automated Electronic Health Record to Electronic Data Capture Transfer in Clinical Studies in the German Health Care System: Feasibility Study and Gap Analysis
Source: J Med Internet Res. 2023 Aug 4;25:e47958. doi: 10.2196/47958 (PMC10439471; doi:10.2196/47958)
Supplement: Multimedia Appendix 1 [file jmir_v25i1e47958_app1.pdf]

**Multimedia Appendix – Comorbidities by ICD-10 codes:**

**Acute Coronary Syndrome**

| ICD-10 | ICD-10 Decode |
|---|---|
| I24 | Other acute ischemic heart diseases |
| I24.0 | Acute coronary thrombosis not resulting in myocardial infarction |
| I24.1 | Dressler's syndrome |
| I24.8 | Other forms of acute ischemic heart disease |
| I24.9 | Acute ischemic heart disease, unspecified |
| I20 | Angina pectoris |
| I20.0 | Unstable angina |
| I20.1 | Angina pectoris with documented spasm |
| I20.2 | Refractory angina pectoris |
| I20.8 | Other forms of angina pectoris |
| I20.9 | Angina pectoris, unspecified |

**Alcohol consumption**

| ICD-10 | ICD-10 Decode |
|---|---|
| F10.2 | Alcohol dependence |
| F10.20 | Alcohol dependence, uncomplicated |
| F10.21 | Alcohol dependence, in remission |
| F10.22 | Alcohol dependence with intoxication |
| F10.22 | Alcohol dependence with intoxication, uncomplicated |
| F10.221 | Alcohol dependence with intoxication delirium |
| F10.229 | Alcohol dependence with intoxication, unspecified |
| F10.23 | Alcohol dependence with withdrawal |
| F10.230 | Alcohol dependence with withdrawal, uncomplicated |
| F10.230 | Alcohol dependence with withdrawal delirium |
| F10.232 | Alcohol dependence with withdrawal with perceptual disturbance |
| F10.239 | Alcohol dependence with withdrawal, unspecified |
| F10.24 | Alcohol dependence with alcohol-induced mood disorder |
| F10.25 | Alcohol dependence with alcohol-induced psychotic disorder |
| F10.250 | Alcohol dependence with alcohol-induced psychotic disorder with delusions |
| F10.251 | Alcohol dependence with alcohol-induced psychotic disorder with hallucinations |
| F10.259 | Alcohol dependence with alcohol-induced psychotic disorder, unspecified |
| F10.26 | Alcohol dependence with alcohol-induced persisting amnestic disorder |
| F10.27 | Alcohol dependence with alcohol-induced persisting dementia |
| F10.28 | Alcohol dependence with other alcohol-induced disorders |
| F10.280 | Alcohol dependence with alcohol-induced anxiety disorder |
| F10.281 | Alcohol dependence with alcohol-induced sexual dysfunction |

| | |
|---|---|
| F10.282 | Alcohol dependence with alcohol-induced sleep disorder |
| F10.288 | Alcohol dependence with other alcohol-induced disorder |
| F10.29 | Alcohol dependence with unspecified alcohol-induced disorder |
| F10.90 | Alcohol use, unspecified, uncomplicated |
| F10.91 | Alcohol use, unspecified, in remission |
| F10.92 | Alcohol use, unspecified with intoxication |
| F10.920 | Alcohol use, unspecified with intoxication, uncomplicated |
| F10.921 | Alcohol use, unspecified with intoxication delirium |
| F10.929 | Alcohol use, unspecified with intoxication, unspecified |
| F10.93 | Alcohol use, unspecified with withdrawal |
| F10.930 | Alcohol use, unspecified with withdrawal, uncomplicated |
| F10.931 | Alcohol use, unspecified with withdrawal delirium |
| F10.932 | Alcohol use, unspecified with withdrawal with perceptual disturbance |
| F10.939 | Alcohol use, unspecified with withdrawal, unspecified |
| F10.94 | Alcohol use, unspecified with alcohol-induced mood disorder |
| F10.95 | Alcohol use, unspecified with alcohol-induced psychotic disorder |
| F10.950 | Alcohol use, unspecified with alcohol-induced psychotic disorder with delusions |
| F10.951 | Alcohol use, unspecified with alcohol-induced psychotic disorder with hallucinations |
| F10.959 | Alcohol use, unspecified with alcohol-induced psychotic disorder, unspecified |
| F10.96 | Alcohol use, unspecified with alcohol-induced persisting amnestic disorder |
| F10.97 | Alcohol use, unspecified with alcohol-induced persisting dementia |
| F10.98 | Alcohol use, unspecified with other alcohol-induced disorders |
| F10.980 | Alcohol use, unspecified with alcohol-induced anxiety disorder |
| F10.981 | Alcohol use, unspecified with alcohol-induced sexual dysfunction |
| F10.982 | Alcohol use, unspecified with alcohol-induced sleep disorder |
| F10.988 | Alcohol use, unspecified with other alcohol-induced disorder |
| F10.99 | Alcohol use, unspecified with unspecified alcohol-induced disorder |
| Z71.41 | Alcohol abuse counseling and surveillance of alcoholic |
| K70 | Alcoholic liver disease |
| K70.0 | Alcoholic fatty liver |
| K70.1 | Alcoholic hepatitis |
| K70.10 | Alcoholic hepatitis without ascites |
| K70.11 | Alcoholic hepatitis with ascites |
| K70.2 | Alcoholic fibrosis and sclerosis of liver |
| K70.3 | Alcoholic cirrhosis of liver |
| K70.30 | Alcoholic cirrhosis of liver without ascites |
| K70.31 | Alcoholic cirrhosis of liver with ascites |
| K70.4 | Alcoholic hepatic failure |
| K70.40 | Alcoholic hepatic failure without coma |
| K70.41 | Alcoholic hepatic failure with coma |
| K70.9 | Alcoholic liver disease, unspecified |
| G31.2 | Degeneration of nervous system due to alcohol |

| | |
|---|---|
| I42.6 | Alcoholic cardiomyopathy |
| Z71.4 | Alcohol abuse counseling and surveillance |
| Z71.41 | Alcohol abuse counseling and surveillance of alcoholic |
| Z71.42 | Counseling for family member of alcoholic |
| K29.2 | Alcoholic gastritis |
| K29.20 | Alcoholic gastritis without bleeding |
| K29.21 | Alcoholic gastritis with bleeding |
| G72.1 | Alcoholic myopathy |
| G62.1 | Alcoholic polyneuropathy |
| K85.2 | Alcohol induced acute pancreatitis |
| K85.20 | Alcohol induced acute pancreatitis without necrosis or infection |
| K85.21 | Alcohol induced acute pancreatitis with uninfected necrosis |
| K85.22 | Alcohol induced acute pancreatitis with infected necrosis |
| E24.4 | Alcohol-induced pseudo-Cushing's syndrome |

**Ascites:**

| ICD-10 | ICD-10 Decode |
|---|---|
| R18 | Ascites |
| R18.0 | Malignant ascites |
| R18.8 | Other ascites |
| R60.0 | Localized edema |
| R60.9 | Edema, unspecified |
| K70.31 | Alcoholic cirrhosis of liver with ascites |
| K70.11 | Alcoholic hepatitis with ascites |
| K71.51 | Toxic liver disease with chronic active hepatitis with ascites |

**Atrial fibrillation:**

| ICD-10 | ICD-10 Decode |
|---|---|
| I48.0 | Paroxysmal atrial fibrillation |
| I48.1 | Persistent atrial fibrillation |
| I48.2 | Chronic atrial fibrillation |
| I48.20 | Chronic atrial fibrillation, unspecified |
| I48.21 | Permanent atrial fibrillation |
| I48.91 | Unspecified atrial fibrillation |

**Cardiogenic shock:**

| ICD-10 | ICD-10 Decode |
|---|---|
| R57.0 | Cardiogenic shock |
| T81.11 | Postprocedural cardiogenic shock |
| R57.1 | Hypovolemic shock |

| | |
|---|---|
| R57.8 | Other shock |
| R57.9 | Shock, unspecified |

**Chronic heart failure**

| ICD-10 | ICD-10 Decode |
|---|---|
| I50.- | Heart failure |
| I50.0- | Congestive heart failure |
| I50.00 | Primary congestive heart failure |
| I50.01 | Secondary congestive heart failure |
| I50.02! | Congestive heart failure, asymptomatic |
| I50.03! | Congestive heart failure with symptoms under severe strain |
| I50.04! | Congestive heart failure with symptoms under mild strain |
| I50.05! | Congestive heart failure with symptoms at rest |
| I50.1- | Left ventricular failure, unspecified |
| I50.11 | Left ventricular failure: Asymptomatic |
| I50.12 | Left ventricular failure: With symptoms under severe strain |
| I50.13 | Left ventricular failure: With symptoms under mild strain |
| I50.14 | Left ventricular failure: With symptoms at rest |
| I50.19 | Left ventricular failure: Unspecified |
| I50.9 | Heart failure, unspecified |

**Chronic kidney disease (CKD):**

| ICD-10 | ICD-10 Decode |
|---|---|
| E09.29 | Drug or chemical induced diabetes mellitus with other diabetic kidney complication |
| E08.2 | Diabetes mellitus due to underlying condition with kidney complications |
| E08.21 | Diabetes mellitus due to underlying condition with diabetic nephropathy |
| E08.22 | Diabetes mellitus due to underlying condition with diabetic chronic kidney disease |
| E08.29 | Diabetes mellitus due to underlying condition with other diabetic kidney complication |
| E13.2 | Other specified diabetes mellitus with kidney complications |
| E13.21 | Other specified diabetes mellitus with diabetic nephropathy |
| E13.22 | Other specified diabetes mellitus with diabetic chronic kidney disease |
| E13.29 | Other specified diabetes mellitus with other diabetic kidney complication |
| E11.2 | Type 2 diabetes mellitus with kidney complications |
| E11.21 | Type 2 diabetes mellitus with diabetic nephropathy |
| E11.22 | Type 2 diabetes mellitus with diabetic chronic kidney disease |
| E11.29 | Type 2 diabetes mellitus with other diabetic kidney complication |
| I13 | Hypertensive heart and chronic kidney disease |
| I13.0 | Hypertensive heart and chronic kidney disease with heart failure and stage 1 through stage 4 chronic kidney disease, or unspecified chronic kidney disease |
| I13.1 | Hypertensive heart and chronic kidney disease without heart failure |

| | |
|---|---|
| I13.10 | Hypertensive heart and chronic kidney disease without heart failure, with stage 1 through stage 4 chronic kidney disease, or unspecified chronic kidney disease |
| I13.11 | Hypertensive heart and chronic kidney disease without heart failure, with stage 5 chronic kidney disease, or end stage renal disease |
| I13.2 | Hypertensive heart and chronic kidney disease with heart failure and with stage 5 chronic kidney disease, or end stage renal disease |
| I12 | Hypertensive chronic kidney disease |
| I12.0 | Hypertensive chronic kidney disease with stage 5 chronic kidney disease or end stage renal disease |
| I12.9 | Hypertensive chronic kidney disease with stage 1 through stage 4 chronic kidney disease, or unspecified chronic kidney disease |

**Cigarrette smoking**

| ICD-10 | ICD-10 Decode |
|---|---|
| Z72.0 | Tobacco use |
| F17.2 | Nicotine dependence |
| F17.20 | Nicotine dependence, unspecified |
| F17.200 | Nicotine dependence, unspecified, uncomplicated |
| F17.201 | Nicotine dependence, unspecified, in remission |
| F17.203 | Nicotine dependence unspecified, with withdrawal |
| F17.208 | Nicotine dependence, unspecified, with other nicotine-induced disorders |
| F17.209 | Nicotine dependence, unspecified, with unspecified nicotine-induced disorders |
| F17.21 | Nicotine dependence, cigarettes |
| F17.210 | Nicotine dependence, cigarettes, uncomplicated |
| F17.211 | Nicotine dependence, cigarettes, in remission |
| F17.213 | Nicotine dependence, cigarettes, with withdrawal |
| F17.218 | Nicotine dependence, cigarettes, with other nicotine-induced disorders |
| F17.219 | Nicotine dependence, cigarettes, with unspecified nicotine-induced disorders |
| F17.29 | Nicotine dependence, other tobacco product |
| F17.290 | Nicotine dependence, other tobacco product, uncomplicated |
| F17.291 | Nicotine dependence, other tobacco product, in remission |
| F17.293 | Nicotine dependence, other tobacco product, with withdrawal |
| F17.298 | Nicotine dependence, other tobacco product, with other nicotine-induced disorders |
| F17.299 | Nicotine dependence, other tobacco product, with unspecified nicotine-induced disorders |
| Z87.891 | Personal history of nicotine dependence |
| Z81.2 | Family history of tobacco abuse and dependence |
| Z71.6 | Tobacco abuse counseling |

**Claudication:**

| ICD-10 | ICD-10 Decode |
|---|---|

| | |
|---|---|
| I70.2 | Atherosclerosis of native arteries of the extremities |
| I70.20 | Unspecified atherosclerosis of native arteries of extremities |
| I70.201 | Unspecified atherosclerosis of native arteries of extremities, right leg |
| I70.202 | Unspecified atherosclerosis of native arteries of extremities, left leg |
| I70.203 | Unspecified atherosclerosis of native arteries of extremities, bilateral legs |
| I70.208 | Unspecified atherosclerosis of native arteries of extremities, other extremity |
| I70.209 | Unspecified atherosclerosis of native arteries of extremities, unspecified extremity |
| I70.21 | Atherosclerosis of native arteries of extremities with intermittent claudication |
| I70.211 | Atherosclerosis of native arteries of extremities with intermittent claudication, right leg |
| I70.212 | Atherosclerosis of native arteries of extremities with intermittent claudication, left leg |
| I70.213 | Atherosclerosis of native arteries of extremities with intermittent claudication, bilateral legs |
| I70.218 | Atherosclerosis of native arteries of extremities with intermittent claudication, other extremity |
| I70.219 | Atherosclerosis of native arteries of extremities with intermittent claudication, unspecified extremity |
| I70.29 | Other atherosclerosis of native arteries of extremities |
| I70.291 | Other atherosclerosis of native arteries of extremities, right leg |
| I70.292 | Other atherosclerosis of native arteries of extremities, left leg |
| I70.293 | Other atherosclerosis of native arteries of extremities, bilateral legs |
| I70.298 | Other atherosclerosis of native arteries of extremities, other extremity |
| I70.299 | Other atherosclerosis of native arteries of extremities, unspecified extremity |
| I70.8 | Atherosclerosis of other arteries |
| I70.9 | Other and unspecified atherosclerosis |
| I70.90 | Unspecified atherosclerosis |
| I70.91 | Generalized atherosclerosis |
| I70.92 | Chronic total occlusion of artery of the extremities |

**Diabetes mellitus Type 2:**

| **ICD-10** | **ICD-10 Decode** |
|---|---|
| E11 | Type 2 diabetes mellitus |
| E11.0 | Type 2 diabetes mellitus with hyperosmolarity |
| E11.01 | Type 2 diabetes mellitus with hyperosmolarity with coma |
| E11.1 | Type 2 diabetes mellitus with ketoacidosis |
| E11.11 | Type 2 diabetes mellitus with ketoacidosis with coma |
| E11.2 | Type 2 diabetes mellitus with kidney complications |
| E11.20 | Type 2 diabetes mellitus with renal complications Controlled |
| E11.21 | Type 2 diabetes mellitus with diabetic nephropathy |
| E11.3 | Type 2 diabetes mellitus with ophthalmic complications |
| E11.30 | Type 2 diabetes mellitus with ophthalmic complications Controlled |
| E11.31 | Type 2 diabetes mellitus with unspecified diabetic retinopathy |
| E11.4 | Type 2 diabetes mellitus with neurological complications |

| | |
|---|---|
| E11.40 | Type 2 diabetes mellitus with diabetic neuropathy, unspecified |
| E11.41 | Type 2 diabetes mellitus with diabetic mononeuropathy |
| E11.5 | Type 2 diabetes mellitus with circulatory complications |
| E11.50 | Type 2 diabetes mellitus with peripheral circulatory complications Controlled |
| E11.51 | Type 2 diabetes mellitus with diabetic peripheral angiopathy without gangrene |
| E11.6 | Type 2 diabetes mellitus with other specified complications |
| E11.60 | Type 2 diabetes mellitus with other specified complications Controlled |
| E11.61 | Type 2 diabetes mellitus with diabetic arthropathy |
| E11.7 | Type 2 diabetes mellitus with multiple complications |
| E11.72 | Type 2 diabetes mellitus with multiple complications with other multiple complications, controlled |
| E11.73 | Type 2 diabetes mellitus with multiple complications with other multiple complications, uncontrolled |
| E11.74 | Type 2 diabetes mellitus with multiple complications with diabetic foot syndrome, controlled |
| E11.75 | Type 2 diabetes mellitus with multiple complications with diabetic foot syndrome, uncontrolled |
| E11.8 | Type 2 diabetes mellitus with unspecified complications |
| E11.80 | Type 2 diabetes mellitus with unspecified complications Controlled |
| E11.81 | Type 2 diabetes mellitus with unspecified complications Uncontrolled |
| E11.9 | Type 2 diabetes mellitus without complications |
| E11.90 | Type 2 diabetes mellitus without complications Controlled |
| E11.91 | Type 2 diabetes mellitus without complications Uncontrolled |

**Diabetic retinopathy:**

| ICD-10 | ICD-10 Decode |
|---|---|
| H36.0* | Diabetic retinopathy |
| E11.31 | Type 2 diabetes mellitus with unspecified diabetic retinopathy |
| E11.32 | Type 2 diabetes mellitus with mild nonproliferative diabetic retinopathy |
| E11.33 | Type 2 diabetes mellitus with moderate nonproliferative diabetic retinopathy |
| E11.34 | Type 2 diabetes mellitus with severe nonproliferative diabetic retinopathy |
| E11.35 | Type 2 diabetes mellitus with proliferative diabetic retinopathy |

**Dyspnea:**

| ICD-10 | ICD-10 Decode |
|---|---|
| R06.0 | Dyspnea |
| R06.00 | Dyspnea, unspecified |
| R06.01 | Orthopnea |
| R06.02 | Shortness of breath |
| R06.09 | Other forms of dyspnea |

| | |
|---|---|
| R06.- | Abnormalities of breathing |
| R06.2 | Wheezing |
| R06.3 | Periodic breathing |
| R06.5 | Mouth breathing |
| R06.8- | Other abnormalities of breathing |
| R06.88 | Other and unspecified abnormalities of breathing |
| R07.1 | Chest pain on breathing |
| R09.- | Other symptoms and signs involving the circulatory and respiratory system |
| R09.8 | Other specified symptoms and signs involving the circulatory and respiratory systems |

**Fatigue:**

| **ICD-10** | **ICD-10 Decode** |
|---|---|
| R53.1 | Weakness |
| R53.82 | Chronic fatigue, unspecified |
| R53.83 | Other fatigue |

**Hyperkalemia:**

| **ICD-10** | **ICD-10 Decode** |
|---|---|
| E87.6 | Hyperkalaemia |

**Hypertension:**

| **ICD-10** | **ICD-10 Decode** |
|---|---|
| I15.80 | Other secondary hypertension Without mention of hypertensive urgency |
| I15.81 | Other secondary hypertension With mention of hypertensive urgency |
| I15.9 | Secondary hypertension, unspecified |
| I15.90 | Secondary hypertension, unspecified Without mention of hypertensive urgency |
| I15.91 | Secondary hypertension, unspecified With mention of hypertensive urgency |

**Limb ischemia:**

| **ICD-10** | **ICD-10 Decode** |
|---|---|
| I70.2 | Atherosclerosis of native arteries of the extremities |
| I70.20 | Unspecified atherosclerosis of native arteries of extremities |
| I70.201 | Unspecified atherosclerosis of native arteries of extremities, right leg |
| I70.202 | Unspecified atherosclerosis of native arteries of extremities, left leg |
| I70.203 | Unspecified atherosclerosis of native arteries of extremities, bilateral legs |
| I70.208 | Unspecified atherosclerosis of native arteries of extremities, other extremity |
| I70.209 | Unspecified atherosclerosis of native arteries of extremities, unspecified extremity |

| | |
|---|---|
| I70.29 | Other atherosclerosis of native arteries of extremities |
| I70.291 | Other atherosclerosis of native arteries of extremities, right leg |
| I70.292 | Other atherosclerosis of native arteries of extremities, left leg |
| I70.293 | Other atherosclerosis of native arteries of extremities, bilateral legs |
| I70.298 | Other atherosclerosis of native arteries of extremities, other extremity |
| I70.299 | Other atherosclerosis of native arteries of extremities, unspecified extremity |
| I70.8 | Atherosclerosis of other arteries |
| I70.9 | Other and unspecified atherosclerosis |
| I70.90 | Unspecified atherosclerosis |
| I70.91 | Generalized atherosclerosis |
| I70.92 | Chronic total occlusion of artery of the extremities |
| I70.22 | Atherosclerosis of native arteries of extremities with rest pain |
| I70.221 | Atherosclerosis of native arteries of extremities with rest pain, right leg |
| I70.222 | Atherosclerosis of native arteries of extremities with rest pain, left leg |
| I70.223 | Atherosclerosis of native arteries of extremities with rest pain, bilateral legs |
| I70.228 | Atherosclerosis of native arteries of extremities with rest pain, other extremity |
| I70.229 | Atherosclerosis of native arteries of extremities with rest pain, unspecified extremity |
| I70.26 | Atherosclerosis of native arteries of extremities with gangrene |
| I70.261 | Atherosclerosis of native arteries of extremities with gangrene, right leg |
| I70.262 | Atherosclerosis of native arteries of extremities with gangrene, left leg |
| I70.263 | Atherosclerosis of native arteries of extremities with gangrene, bilateral legs |
| I70.268 | Atherosclerosis of native arteries of extremities with gangrene, other extremity |
| I70.269 | Atherosclerosis of native arteries of extremities with gangrene, unspecified extremity |

**Myocardial infarction:**

| **ICD-10** | **ICD-10 Decode** |
|---|---|
| I23.0 | Hemopericardium as current complication following acute myocardial infarction |
| I23.1 | Atrial septal defect as current complication following acute myocardial infarction |
| I23.2 | Ventricular septal defect as current complication following acute myocardial infarction |
| I23.3 | Rupture of cardiac wall without hemopericardium as current complication following acute myocardial infarction |
| I23.4 | Rupture of chordae tendineae as current complication following acute myocardial infarction |
| I23.5 | Rupture of papillary muscle as current complication following acute myocardial infarction |
| I23.6 | Thrombosis of atrium, auricular appendage, and ventricle as current complications following acute myocardial infarction |
| I23.7 | Postinfarction angina |
| I23.8 | Other current complications following acute myocardial infarction |
| I25.2 | Old myocardial infarction |
| I25.20 | Old myocardial infarction: 29 days to under 4 months previously |

| | |
|---|---|
| I25.21 | Old myocardial infarction: 4 days to under 1 year previously |
| I25.22 | Old myocardial infarction: 1 year or more previously |
| I25.29 | Old myocardial infarction: Unspecified |
| I25.5 | Ischemic cardiomyopathy |
| I25.6 | Silent myocardial ischemia |
| I25.83 | Coronary atherosclerosis due to lipid rich plaque |
| I25.84 | Coronary atherosclerosis due to calcified coronary lesion |
| I25.89 | Other forms of chronic ischemic heart disease |
| I25.9 | Chronic ischemic heart disease, unspecified |
| Z03.4 | Observation for suspected myocardial infarction |

**Peripheral edema:**

| ICD-10 | ICD-10 Decode |
|---|---|
| R60.0 | Localized edema |
| R60.1 | Generalized edema |
| R60.9 | Edema, unspecified |

**Pleural effusion:**

| ICD-10 | ICD-10 Decode |
|---|---|
| J90 | Pleural effusion, not elsewhere classified |
| J91 | Pleural effusion in conditions classified elsewhere |
| J91.0 | Malignant pleural effusion |
| J91.8 | Pleural effusion in other conditions classified elsewhere |
| J94.0 | Chylous effusion |
| J94.9 | Pleural condition, unspecified |

**Pregnancy:**

| ICD-10 | ICD-10 Decode |
|---|---|
| Z30 | Encounter for contraceptive management |
| Z32 | Encounter for pregnancy test and childbirth and childcare instruction |
| Z33! | Pregnant state |
| Z34 | Encounter for supervision of normal pregnancy |

**Sepsis:**

| ICD 10 | ICD 10 Decode |
|---|---|
| A02.1 | Salmonella sepsis |

| | |
|---|---|
| A20.7 | Septicaemic plague |
| A21.7 | Generalized tularaemia |
| A22.7 | Anthrax sepsis |
| A26.7 | Erysipelothrix sepsis |
| A32.7 | Listerial sepsis |
| A39.2 | Acute meningococcaemia |
| A39.3 | Chronic meningococcaemia |
| A39.4 | Meningococcaemia, unspecified |
| A40 | Streptococcal sepsis |
| A40.0 | Sepsis due to streptococcus, group A |
| A40.1 | Sepsis due to streptococcus, group B |
| A40.2 | Sepsis due to streptococcus, group D and enterococcus |
| A40.3 | Sepsis due to Streptococcus pneumoniae |
| A40.8 | Other streptococcal sepsis |
| A40.9 | Streptococcal sepsis, unspecified |
| A41 | Other sepsis |
| A41.0 | Sepsis due to Staphylococcus aureus |
| A41.1 | Sepsis due to other specified staphylococcus |
| A41.2 | Sepsis due to unspecified staphylococcus |
| A41.3 | Sepsis due to Haemophilus influenzae |
| A41.4 | Sepsis due to anaerobes |
| A41.5 | Sepsis due to other Gram-negative organisms |
| A41.51 | Sepsis: Escherichia coli [E. coli] |
| A41.52 | Sepsis: Pseudomonas |
| A41.58 | Sepsis: Other gramnegative pathogen |
| A41.8 | Other specified sepsis |
| A41.9 | Sepsis, unspecified |
| A42.7 | Actinomycotic sepsis |
| A48.3 | Toxic shock syndrome |
| B00.70 | Herpesviral sepsis |
| B37.7 | Candidal sepsis |
| B38.70 | Disseminated coccidioidomycosis |
| B39.30 | Disseminated histoplasmosis capsulati |
| B40.70 | Disseminated blastomycosis |
| B41.70 | Disseminated paracoccidioidomycosis |
| B42.70 | Disseminated sporotrichosis |
| B44.70 | Disseminated aspergillosis |
| B45.70 | Disseminated cryptococcosis |
| B46.40 | Disseminated mucormycosis |
| B48.80 | Lobomycosis |
| B58.90 | Toxoplasmosis, unspecified |
| B60.80 | Other specified protozoal diseases |

| | |
|---|---|
| U69.8 | Secondary code numbers specifying the temporal relationship of sepsis and septic shock to inpatient hospital admission |
| U69.80 | non-nosocomial Sepsis |
| U69.81 | nosocomial Sepsis |
| U69.82 | Sepsis of unclear time of onset related to hospital admission |
| R57.2 | Septic shock |

**Stroke:**

| ICD-10 | ICD-10 Decode |
|---|---|
| I63 | Cerebral infarction |
| I63.0 | Cerebral infarction due to thrombosis of precerebral arteries |
| I63.00 | Cerebral infarction due to thrombosis of unspecified precerebral artery |
| I63.01 | Cerebral infarction due to thrombosis of vertebral artery |
| I63.011 | Cerebral infarction due to thrombosis of right vertebral artery |
| I63.012 | Cerebral infarction due to thrombosis of left vertebral artery |
| I63.013 | Cerebral infarction due to thrombosis of bilateral vertebral arteries |
| I63.019 | Cerebral infarction due to thrombosis of unspecified vertebral artery |
| I63.02 | Cerebral infarction due to thrombosis of basilar artery |
| I63.03 | Cerebral infarction due to thrombosis of carotid artery |
| I63.031 | Cerebral infarction due to thrombosis of right carotid artery |
| I63.032 | Cerebral infarction due to thrombosis of left carotid artery |
| I63.033 | Cerebral infarction due to thrombosis of bilateral carotid arteries |
| I63.039 | Cerebral infarction due to thrombosis of unspecified carotid artery |
| I63.09 | Cerebral infarction due to thrombosis of other precerebral artery |
| I63.1 | Cerebral infarction due to embolism of precerebral arteries |
| I63.10 | Cerebral infarction due to embolism of unspecified precerebral artery |
| I63.11 | Cerebral infarction due to embolism of vertebral artery |
| I63.111 | Cerebral infarction due to embolism of right vertebral artery |
| I63.112 | Cerebral infarction due to embolism of left vertebral artery |
| I63.113 | Cerebral infarction due to embolism of bilateral vertebral arteries |
| I63.119 | Cerebral infarction due to embolism of unspecified vertebral artery |
| I63.12 | Cerebral infarction due to embolism of basilar artery |
| I63.13 | Cerebral infarction due to embolism of carotid artery |
| I63.131 | Cerebral infarction due to embolism of right carotid artery |
| I63.132 | Cerebral infarction due to embolism of left carotid artery |
| I63.133 | Cerebral infarction due to embolism of bilateral carotid arteries |
| I63.139 | Cerebral infarction due to embolism of unspecified carotid artery |
| I63.19 | Cerebral infarction due to embolism of other precerebral artery |
| I63.2 | Cerebral infarction due to unspecified occlusion or stenosis of precerebral arteries |
| I63.20 | Cerebral infarction due to unspecified occlusion or stenosis of unspecified precerebral arteries |
| I63.21 | Cerebral infarction due to unspecified occlusion or stenosis of vertebral arteries |

| | |
|---|---|
| I63.211 | Cerebral infarction due to unspecified occlusion or stenosis of right vertebral artery |
| I63.212 | Cerebral infarction due to unspecified occlusion or stenosis of left vertebral artery |
| I63.213 | Cerebral infarction due to unspecified occlusion or stenosis of bilateral vertebral arteries |
| I63.219 | Cerebral infarction due to unspecified occlusion or stenosis of unspecified vertebral artery |
| I63.22 | Cerebral infarction due to unspecified occlusion or stenosis of basilar artery |
| I63.23 | Cerebral infarction due to unspecified occlusion or stenosis of carotid arteries |
| I63.231 | Cerebral infarction due to unspecified occlusion or stenosis of right carotid arteries |
| I63.232 | Cerebral infarction due to unspecified occlusion or stenosis of left carotid arteries |
| I63.233 | Cerebral infarction due to unspecified occlusion or stenosis of bilateral carotid arteries |
| I63.239 | Cerebral infarction due to unspecified occlusion or stenosis of unspecified carotid artery |
| I63.29 | Cerebral infarction due to unspecified occlusion or stenosis of other precerebral arteries |
| I63.3 | Cerebral infarction due to thrombosis of cerebral arteries |
| I63.30 | Cerebral infarction due to thrombosis of unspecified cerebral artery |
| I63.31 | Cerebral infarction due to thrombosis of middle cerebral artery |
| I63.311 | Cerebral infarction due to thrombosis of right middle cerebral artery |
| I63.312 | Cerebral infarction due to thrombosis of left middle cerebral artery |
| I63.313 | Cerebral infarction due to thrombosis of bilateral middle cerebral arteries |
| I63.319 | Cerebral infarction due to thrombosis of unspecified middle cerebral artery |
| I63.32 | Cerebral infarction due to thrombosis of anterior cerebral artery |
| I63.321 | Cerebral infarction due to thrombosis of right anterior cerebral artery |
| I63.322 | Cerebral infarction due to thrombosis of left anterior cerebral artery |
| I63.323 | Cerebral infarction due to thrombosis of bilateral anterior cerebral arteries |
| I63.329 | Cerebral infarction due to thrombosis of unspecified anterior cerebral artery |
| I63.33 | Cerebral infarction due to thrombosis of posterior cerebral artery |
| I63.331 | Cerebral infarction due to thrombosis of right posterior cerebral artery |
| I63.332 | Cerebral infarction due to thrombosis of left posterior cerebral artery |
| I63.333 | Cerebral infarction due to thrombosis of bilateral posterior cerebral arteries |
| I63.339 | Cerebral infarction due to thrombosis of unspecified posterior cerebral artery |
| I63.34 | Cerebral infarction due to thrombosis of cerebellar artery |
| I63.341 | Cerebral infarction due to thrombosis of right cerebellar artery |
| I63.342 | Cerebral infarction due to thrombosis of left cerebellar artery |
| I63.343 | Cerebral infarction due to thrombosis of bilateral cerebellar arteries |
| I63.349 | Cerebral infarction due to thrombosis of unspecified cerebellar artery |
| I63.39 | Cerebral infarction due to thrombosis of other cerebral artery |
| I63.4 | Cerebral infarction due to embolism of cerebral arteries |
| I63.40 | Cerebral infarction due to embolism of unspecified cerebral artery |
| I63.41 | Cerebral infarction due to embolism of middle cerebral artery |
| I63.411 | Cerebral infarction due to embolism of right middle cerebral artery |

| | |
|---|---|
| I63.412 | Cerebral infarction due to embolism of left middle cerebral artery |
| I63.413 | Cerebral infarction due to embolism of bilateral middle cerebral arteries |
| I63.419 | Cerebral infarction due to embolism of unspecified middle cerebral artery |
| I63.42 | Cerebral infarction due to embolism of anterior cerebral artery |
| I63.421 | Cerebral infarction due to embolism of right anterior cerebral artery |
| I63.422 | Cerebral infarction due to embolism of left anterior cerebral artery |
| I63.423 | Cerebral infarction due to embolism of bilateral anterior cerebral arteries |
| I63.429 | Cerebral infarction due to embolism of unspecified anterior cerebral artery |
| I63.43 | Cerebral infarction due to embolism of posterior cerebral artery |
| I63.431 | Cerebral infarction due to embolism of right posterior cerebral artery |
| I63.432 | Cerebral infarction due to embolism of left posterior cerebral artery |
| I63.433 | Cerebral infarction due to embolism of bilateral posterior cerebral arteries |
| I63.439 | Cerebral infarction due to embolism of unspecified posterior cerebral artery |
| I63.44 | Cerebral infarction due to embolism of cerebellar artery |
| I63.441 | Cerebral infarction due to embolism of right cerebellar artery |
| I63.442 | Cerebral infarction due to embolism of left cerebellar artery |
| I63.443 | Cerebral infarction due to embolism of bilateral cerebellar arteries |
| I63.449 | Cerebral infarction due to embolism of unspecified cerebellar artery |
| I63.49 | Cerebral infarction due to embolism of other cerebral artery |
| I63.5 | Cerebral infarction due to unspecified occlusion or stenosis of cerebral arteries |
| I63.50 | Cerebral infarction due to unspecified occlusion or stenosis of unspecified cerebral artery |
| I63.51 | Cerebral infarction due to unspecified occlusion or stenosis of middle cerebral artery |
| I63.511 | Cerebral infarction due to unspecified occlusion or stenosis of right middle cerebral artery |
| I63.512 | Cerebral infarction due to unspecified occlusion or stenosis of left middle cerebral artery |
| I63.513 | Cerebral infarction due to unspecified occlusion or stenosis of bilateral middle cerebral arteries |
| I63.519 | Cerebral infarction due to unspecified occlusion or stenosis of unspecified middle cerebral artery |
| I63.52 | Cerebral infarction due to unspecified occlusion or stenosis of anterior cerebral artery |
| I63.521 | Cerebral infarction due to unspecified occlusion or stenosis of right anterior cerebral artery |
| I63.522 | Cerebral infarction due to unspecified occlusion or stenosis of left anterior cerebral artery |
| I63.523 | Cerebral infarction due to unspecified occlusion or stenosis of bilateral anterior cerebral arteries |
| I63.529 | Cerebral infarction due to unspecified occlusion or stenosis of unspecified anterior cerebral artery |
| I63.53 | Cerebral infarction due to unspecified occlusion or stenosis of posterior cerebral artery |

| | |
|---|---|
| I63.531 | Cerebral infarction due to unspecified occlusion or stenosis of right posterior cerebral artery |
| I63.532 | Cerebral infarction due to unspecified occlusion or stenosis of left posterior cerebral artery |
| I63.533 | Cerebral infarction due to unspecified occlusion or stenosis of bilateral posterior cerebral arteries |
| I63.539 | Cerebral infarction due to unspecified occlusion or stenosis of unspecified posterior cerebral artery |
| I63.54 | Cerebral infarction due to unspecified occlusion or stenosis of cerebellar artery |
| I63.541 | Cerebral infarction due to unspecified occlusion or stenosis of right cerebellar artery |
| I63.542 | Cerebral infarction due to unspecified occlusion or stenosis of left cerebellar artery |
| I63.543 | Cerebral infarction due to unspecified occlusion or stenosis of bilateral cerebellar arteries |
| I63.549 | Cerebral infarction due to unspecified occlusion or stenosis of unspecified cerebellar artery |
| I63.59 | Cerebral infarction due to unspecified occlusion or stenosis of other cerebral artery |
| I63.6 | Cerebral infarction due to cerebral venous thrombosis, nonpyogenic |
| I63.8 | Other cerebral infarction |
| I63.81 | Other cerebral infarction due to occlusion or stenosis of small artery |
| I63.89 | Other cerebral infarction |
| I63.9 | Cerebral infarction, unspecified |
| G46.3 | Brain stem stroke syndrome |
| G46.4 | Cerebellar stroke syndrome |
| G46.5 | Pure motor lacunar syndrome |
| G46.6 | Pure sensory lacunar syndrome |
| G46.7 | Other lacunar syndromes |
| I69 | Sequelae of cerebrovascular disease |
| I69.30 | Unspecified sequelae of cerebral infarction |
| I61 | Nontraumatic intracerebral hemorrhage |
| I61.0 | Nontraumatic intracerebral hemorrhage in hemisphere, subcortical |
| I61.1 | Nontraumatic intracerebral hemorrhage in hemisphere, cortical |
| I61.2 | Nontraumatic intracerebral hemorrhage in hemisphere, unspecified |
| I61.3 | Nontraumatic intracerebral hemorrhage in brain stem |
| I61.4 | Nontraumatic intracerebral hemorrhage in cerebellum |
| I61.5 | Nontraumatic intracerebral hemorrhage, intraventricular |
| I61.6 | Nontraumatic intracerebral hemorrhage, multiple localized |
| I61.8 | Other nontraumatic intracerebral hemorrhage |
| I61.9 | Nontraumatic intracerebral hemorrhage, unspecified |
| I62 | Other and unspecified nontraumatic intracranial hemorrhage |
| Z86.73 | Personal history of transient ischemic attack (TIA), and cerebral infarction without residual deficits |
| R29.7 | National Institutes of Health Stroke Scale (NIHSS) score |

**Supraventricular tachycardia**

| ICD-10 | ICD-10 Decode |
|---|---|
| I47.1 | Supraventricular tachycardia |

**Transient ischemic attack (TIA)**

| | |
|---|---|
| G45 | Transient cerebral ischemic attacks and related syndromes |
| G45.0 | Vertebro-basilar artery syndrome |
| G45.1 | Carotid artery syndrome (hemispheric) |
| G45.2 | Multiple and bilateral precerebral artery syndromes |
| G45.3 | Amaurosis fugax |
| G45.4 | Transient global amnesia |
| G45.8 | Other transient cerebral ischemic attacks and related syndromes |
| G45.9 | Transient cerebral ischemic attack, unspecified |
| G45.92 | Transient cerebral ischaemic attack, unspecified Complete remission after 1 to 24 hours |
| G45.93 | Transient cerebral ischaemic attack, unspecified Complete remission after less than 1 hour |
| G45.99 | Transient cerebral ischaemic attack, unspecified Course of remission unspecified |

**Ulcers of extremities**

| ICD-10 | ICD-10 Decode |
|---|---|
| I70.202 | Unspecified atherosclerosis of native arteries of extremities, left leg |
| I70.203 | Unspecified atherosclerosis of native arteries of extremities, bilateral legs |
| I70.208 | Unspecified atherosclerosis of native arteries of extremities, other extremity |
| I70.209 | Unspecified atherosclerosis of native arteries of extremities, unspecified extremity |
| I70.29 | Other atherosclerosis of native arteries of extremities |
| I70.291 | Other atherosclerosis of native arteries of extremities, right leg |
| I70.292 | Other atherosclerosis of native arteries of extremities, left leg |
| I70.293 | Other atherosclerosis of native arteries of extremities, bilateral legs |
| I70.298 | Other atherosclerosis of native arteries of extremities, other extremity |
| I70.299 | Other atherosclerosis of native arteries of extremities, unspecified extremity |
| I70.8 | Atherosclerosis of other arteries |
| I70.9 | Other and unspecified atherosclerosis |
| I70.90 | Unspecified atherosclerosis |
| I70.91 | Generalized atherosclerosis |
| I70.92 | Chronic total occlusion of artery of the extremities |
| I70.23 | Atherosclerosis of native arteries of right leg with ulceration |
| I70.231 | Atherosclerosis of native arteries of right leg with ulceration of thigh |
| I70.232 | Atherosclerosis of native arteries of right leg with ulceration of calf |
| I70.233 | Atherosclerosis of native arteries of right leg with ulceration of ankle |

| | |
|---|---|
| I70.234 | Atherosclerosis of native arteries of right leg with ulceration of heel and midfoot |
| I70.235 | Atherosclerosis of native arteries of right leg with ulceration of other part of foot |
| I70.238 | Atherosclerosis of native arteries of right leg with ulceration of other part of lower leg |
| I70.239 | Atherosclerosis of native arteries of right leg with ulceration of unspecified site |
| I70.24 | Atherosclerosis of native arteries of left leg with ulceration |
| I70.241 | Atherosclerosis of native arteries of left leg with ulceration of thigh |
| I70.242 | Atherosclerosis of native arteries of left leg with ulceration of calf |
| I70.243 | Atherosclerosis of native arteries of left leg with ulceration of ankle |
| I70.244 | Atherosclerosis of native arteries of left leg with ulceration of heel and midfoot |
| I70.245 | Atherosclerosis of native arteries of left leg with ulceration of other part of foot |
| I70.248 | Atherosclerosis of native arteries of left leg with ulceration of other part of lower leg |
| I70.249 | Atherosclerosis of native arteries of left leg with ulceration of unspecified site |
| I70.25 | Atherosclerosis of native arteries of other extremities with ulceration |
| L97 | Non-pressure chronic ulcer of lower limb, not elsewhere classified |
| I83.0 | Varicose veins of lower extremities with ulcer |
| I83.2 | Varicose veins of lower extremities with both ulcer and inflammation |
| I87.01 | Postthrombotic syndrome with ulcer |

**Urinary tract infection:**

| ICD-10 | ICD-10 Decode |
|---|---|
| N39.0 | Urinary tract infection, site not specified |
